# Mechanisms of Intermittent State Transitions in a Coupled Heterogeneous Oscillator Model of Epilepsy

**DOI:** 10.1186/2190-8567-3-17

**Published:** 2013-08-14

**Authors:** Marc Goodfellow, Paul Glendinning

**Affiliations:** 1 Centre for Interdisciplinary Computational and Dynamical Analysis (CICADA), School of Mathematics, The University of Manchester, Manchester, UK

## Abstract

We investigate the dynamic mechanisms underlying intermittent state transitions in a recently proposed neural mass model of epilepsy. A low dimensional model is constructed, which preserves two key features of the neural mass model, namely (i) coupling between oscillators and (ii) heterogeneous proximity of these oscillators to a bifurcation between distinct limit cycles. We demonstrate that state transitions due to intermittency occur in the abstract model. This suggests that there is a general bifurcation mechanism responsible for this behaviour and that this is independent of the precise form of the evolution equations. Such abstractions of neural mass models allow a deeper insight into underlying dynamic and physiological mechanisms, and also allow the more efficient exploration of large scale brain dynamics in disease.

## 1 Introduction

Epilepsy is a prevalent neurological disorder characterised by the repeated occurrence of pathological brain states known as seizures. Seizures are often accompanied by marked changes in electroencephalogram (EEG) dynamics. A pertinent example is the case of absence epilepsy in which short epochs of high amplitude, slow spike-wave rhythms spontaneously arise from a low amplitude background EEG (see, e.g. [1]). Although the reasons for these dynamic state changes are unknown, mathematical modelling can be used to help our understanding of the epileptic brain by highlighting possible underlying mechanisms. In addition, potential differences between the epileptic and non-epileptic brain can be revealed and seizure abatement or prevention strategies can be explored (see, e.g. [2]). 

Physiologically inspired macroscopic brain models (for example neural mass and neural field models) have often been employed to investigate the causes of epilepsy and seizures [3-8]. In characterising the epileptic brain using these models, one can consider ways in which seizures can sporadically reoccur. In general, three methods have been proposed, namely bifurcations, bistability, and intermittency, with each of these potential dynamic mechanisms receiving both experimental and modelling support [4,5,7,9-11]. In particular, intermittency has been postulated to underlie the generation of absence seizures in experimental animal models [10,12]. A mechanistic neural mass model was recently shown to display intermittent dynamic transitions reminiscent of absence seizures [7,13]. In the model of [7], intermittency arose due to interactions between heterogeneous populations of neurons. However, it remains unclear exactly which properties of this model imbue it with these important dynamics, and whether these properties are specific to the neural mass formalism. A greater understanding of these dynamics will highlight which features of interconnected regions of the brain are potentially responsible for seizure transitions. 

Here, we approach this problem by extracting what we believe to be the fundamental dynamic features of the neural mass model, i.e. those features which, independent of the precise form of the equations, lead to the intermittent behaviour. We then construct a simple, low dimensional system preserving these features and demonstrate that this reduced system displays dynamics similar to those of the neural mass model. This makes a deeper understanding of the mechanisms of intermittent transitions possible, whilst it also shows that the proposed mechanism is dynamically robust. Furthermore, we exploit the reduced dimensionality of our new model to explore the effects of coupling in larger, heterogeneous systems. We propose this model abstraction strategy as a complement to the more detailed neural mass and neural network models for exploring the dynamic mechanisms of epilepsy.

## 2 Methods

### 2.1 Model

Our starting point is the work of [7], in which a model composed of connected neural masses was formulated as a representation of interacting populations of neurons in the cortex (e.g. interacting cortical columns). It was shown that this model displayed intermittency, autonomously switching between low amplitude, comparatively fast (i.e. “alpha band”) oscillations and high amplitude, slow spike-wave oscillations. These dynamics are reminiscent of electrographic recordings from patients with absence epilepsy. In the model, we identify the high amplitude oscillation (ghost of the limit cycle, i.e. “laminar” phase) with seizure EEG and the background EEG state is represented by the global re-injection of the model (i.e. the “turbulent” phase). Both intrinsic parameter values of the neural masses (the nodes of the network) and the connectivity between nodes were important for the observed intermittent dynamics in the model, thus pathological rhythm generation was placed into the context of interacting, heterogeneous regions of tissue. 

Here, we explore the hypothesis that the intermittent dynamics of the neural mass model are due to general dynamic properties, namely (i) the intrinsic dynamic repertoire (bifurcations) of isolated nodes and (ii) the interaction between nodes when they are coupled, and when nodes are heterogeneous. We therefore proceed by engineering a simple deterministic system of ordinary differential equations, which incorporates the features we believe to be important for the observed neural mass model intermittency. We first consider the dynamics of individual nodes, which in the neural mass model were close to a bifurcation between qualitatively different oscillations, with a region of bistability between the two. We therefore begin by constructing a simple two-dimensional non-linear system with these features (see details below). We then introduce global coupling between these nodes and examine the dynamics of the coupled system to test for the emergence of intermittency.

**Fig. 1:**
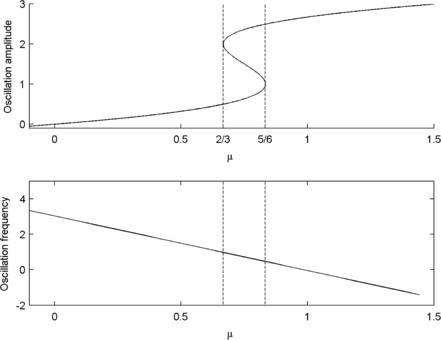
*Model bifurcations.* Bifurcations over *μ* in the single compartment, 2 variable model. *Top*: limit cycle amplitude. *Bottom*: angular velocity

Our simplified model of the neural mass is constructed so that the amplitude of oscillations (*r*) is controlled by a bifurcation parameter, *μ*. Choosing a cubic form for the dependence of $R=r^{2}$ on *μ* and then rotating the system using an angle variable, *θ*, gives rise to the bifurcation structure required, as shown in Fig. 1. The slowing of oscillations with increasing amplitude is obtained by making $\dot{\theta }$ a decreasing function of *R*. In polar coordinates, the model for a single compartment is thus:

(1) $$\begin{array}{rl} \dot{r} & =r\left(\mu -ar^{2}+br^{4}-cr^{6}\right)\\ \dot{\theta } & =\omega -dr^{2} \end{array}$$

To couple *N* different compartments (specified by subscript *i*), we use simple additive coupling, so that in Cartesian coordinates the equations are:

(2) $$\begin{array}{rl} \dot{x_{i}} & =y_{i}\left(\omega -dr_{i}^{2}\right)+x_{i}\left(\mu -ar_{i}^{2}+br_{i}^{4}-cr_{i}^{6}\right)+\beta Ax\\ {\dot{y}}_{i} & =-x_{i}\left(\omega -dr_{i}^{2}\right)+y_{i}\left(\mu -ar_{i}^{2}+br_{i}^{4}-cr_{i}^{6}\right) \end{array}$$

i=1,…,N, where

$$r_{i}^{2}=x_{i}^{2}+y_{i}^{2}$$

The term *A***x** provides the additive coupling, where *A* is the adjacency matrix for a generic network and $x=(x_{1},\ldots ,x_{N})$. As in [7], self-coupling has been included in the intrinsic node equations and, therefore, does not appear explicitly in *A* (i.e. $A_{ii}=0$ ∀*i*).

For convenience, we fix the parameters a=2, b=3/2, and c=1/3. These determine the position of the region of bistability between different oscillation types of the uncoupled system. To see this (and to make sense of future diagrams), we give a brief description of the dynamics of (1). Since $\dot{r}$ is independent of *θ*, stationary points and periodic orbits lie on contours of $\dot{r}=0$, i.e. r=0, the fixed point, which is stable if μ<0 and unstable if μ>0, and the solutions of

$$0=\mu -ar^{2}+br^{4}-cr^{6}$$

which will correspond to a degenerate circle of fixed points if $r^{2}=\omega /d$ is a solution (a special case we ignore), and a periodic orbit otherwise. It is easier to analyze solutions by setting $r^{2}=R$ and looking for *positive* solutions to

$$\mu =aR-bR^{2}+cR^{3}$$

This explains why we have chosen the parameters above: let $F(R)=aR-bR^{2}+cR^{3}$, then

$$F^{\prime }(R)=a-2bR+3cR^{2}$$

and so turning points of this curve (seen as a curve of solutions in the (μ,R)-plane) occur at $F^{\prime }(R)=0$ or, for the parameters a=2, b=3/2, and c=1/3,

$$0=2-3R+R^{2}=(R-2)(R-1)$$

In other words, turning points, which correspond to saddle-node bifurcations of periodic orbits occur at R=2 and R=1 ($r=\sqrt{2}$, r=1). This structure is shown in Fig. 1. If R=2, then $\mu =\frac{2}{3}$, and if R=1, then $\mu =\frac{5}{6}$. A Hopf bifurcation at μ=0 creates a stable low amplitude limit cycle. As *μ* increases through $\mu =\frac{2}{3}$, a second (large amplitude) limit cycle is created together with a large amplitude unstable periodic orbit in a saddle-node bifurcation. Following this bifurcation, there is a region of bistability until $\mu =\frac{5}{6}$ when the stable small amplitude limit cycle is destroyed in the second saddle-node bifurcation. It is this region of bistability that we aim to use in the coupled compartment models to generate intermittency by choosing heterogeneous parameters a little below $\mu =\frac{2}{3}$, so that although each individual compartment lies in a region with a unique stable small amplitude limit cycle, their coupling can cause temporary (but repeated) excitation into the high amplitude state. This is essentially the mechanism proposed in [7]. 

### 2.2 Connectivity

In this study, we explore three different connectivity schemes. In order to demonstrate the equivalence of our model dynamics with those of [7], we initially study an all-to-all coupled system of three compartments (see [13]). For simulations of spatially extended systems, we work in two dimensions with periodic boundaries and symmetric nearest neighbour or distance dependent connectivity. Connectivity weights are scaled by a connectivity parameter, *β*. For systems with distance dependent connectivity, an exponential fall off of connectivity strength is used as follows:

(3) $$a_{ij}=\frac{e^{-\alpha ∥r_{i}-r_{j}∥}}{\max_{ij}(e^{-\alpha ∥r_{i}-r_{j}∥})}$$

where $a_{ij}$ is the entry of *A* connecting nodes *i* and *j* and $r_{i}$ and $r_{j}$ are the 2-d coordinates of the location of compartments *i* and *j*, respectively. The denominator scales the connectivity such that the maximum value of *A* is 1, and this is subsequently scaled in Eq. (2) by *β*.

## 3 Results

### 3.1 Type 1 Intermittency in 3 Coupled Compartments

It has been suggested that the sporadic nature of epileptic episodes in absence seizures is due to dynamic intermittency [10,12]. Our recent work demonstrated that intermittency in heterogeneous neural mass models can arise due to a type 1 route, i.e. a proximal tangent bifurcation of a limit cycle [13,14]. These dynamics arose in a system composed of 3 all-to-all coupled compartments. We therefore investigate whether these dynamics can be recreated in our abstract model, solely based on the generic dynamic features extracted, as described in Sect. 2.

**Fig. 2:**
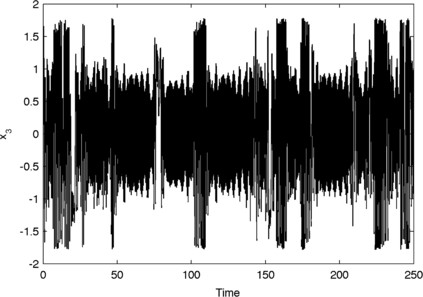
*Intermittency in the abstract model.* Intermittent switching between low amplitude and high amplitude oscillations in variable $x_{3}$ of the model (Eq. (2)) with $\mu_{1}=0.2$, $\mu_{2}=0.3$ and $\mu_{3}=0.6$ and β=3/2

**Fig. 3:**
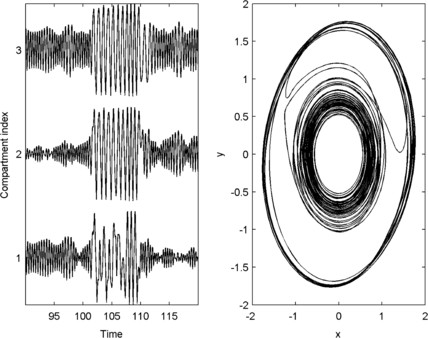
*Intermittent dynamics.Left*: close up of a state transition in the *x*-variable of each of 3 compartments of the model demonstrated in Fig. 1. *Right*: phase portrait showing the trajectory of compartment 3 in *x*–*y* space

In Fig. 2, we demonstrate that intermittency does emerge from our abstract model formulation. Figure 2 shows a long time series with parameter heterogeneity fixed to $\mu_{1}=0.2$, $\mu_{2}=0.3$, and $\mu_{3}=0.6$ and all-to-all connectivity scaled by β=3/2. Each compartment makes intermittent deviations from the low amplitude oscillatory state into a high amplitude, slow oscillation. A close up of these model dynamics can be seen in Fig. 3. 

**Fig. 4:**
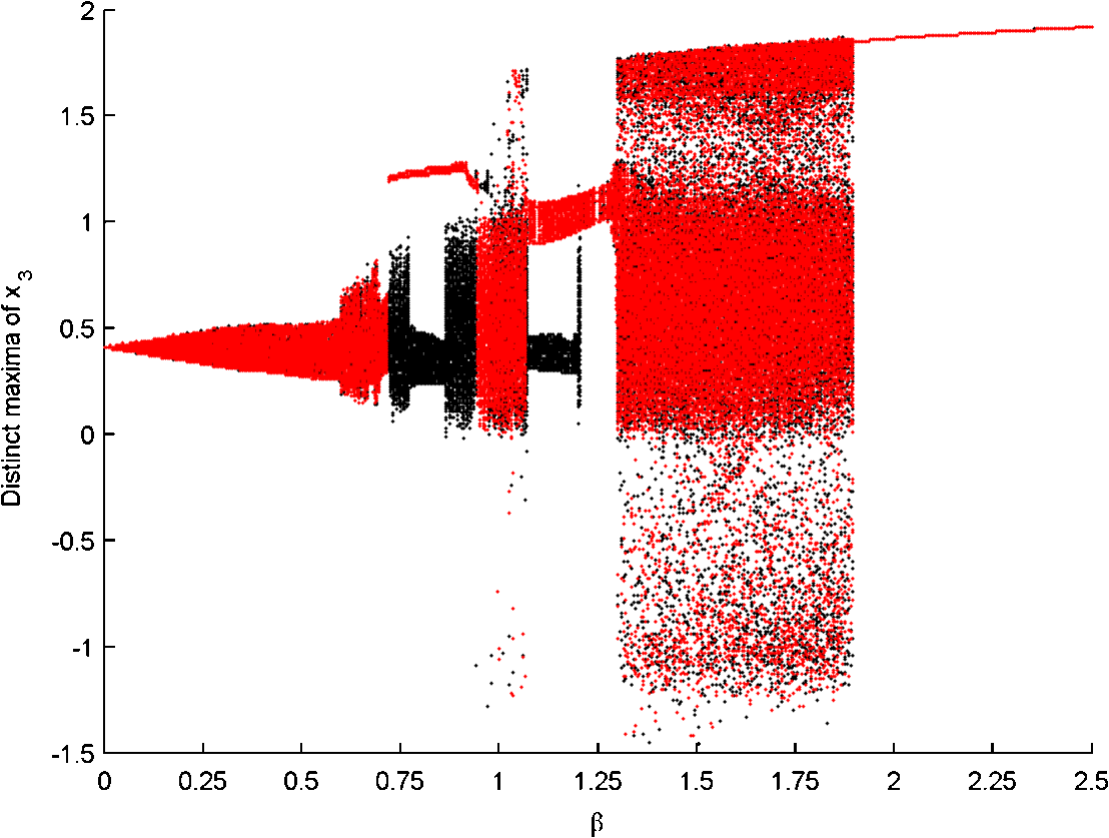
*Effect of coupling strength.* Scan of the dynamics of the 3 compartment model over changes in the coupling strength, *β*. The distinct maxima of $x_{3}$ are shown, with *black* and *red dots* denoting forward and backward scans, respectively. All other parameters are as in Fig. 2

**Fig. 5:**
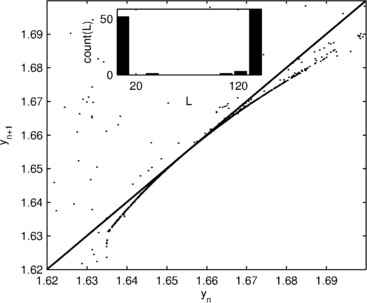
*Type 1 intermittency.The main figure* shows a first return map of variable $y_{1}$ of the system close to the transition into intermittency (β=1.897) and demonstrates the presence of a tangent bifurcation. *The inset* shows the distribution of lengths of the high amplitude states

For fixed $\mu_{i}$ we explored the effect of changing coupling strength, *β*, which is shown in the bifurcation diagram of Fig. 4. For high *β*, the system evolves with synchronous, stable limit cycle oscillations. As coupling strength is decreased these oscillations give way to an intermittent regime, with the system exploring both the high and low amplitude oscillations of the single compartment model. We note that the way in which these model dynamics vary in relation to coupling strength is very similar to the full neural mass model (see [13]). In order to explore the dynamic mechanisms leading to this intermittent window, a long simulation was performed close to the onset of intermittency. Analysis of this system revealed a U-shaped distribution of high amplitude state durations as well as a proximate tangent bifurcation of the phase locked limit cycle (Fig. 5). Thus, our abstract model also follows a type 1 route into intermittency (Chap. 5, pp. 68–69 in [15]), [13]. 

### 3.2 Dynamics of Larger Systems

An advantage of the reduced dimensionality of our new model is the added computational efficiency for simulating large systems. Thus, the role of connectivity and heterogeneity in intermittent state changes can be explored more easily. In this study, we explore the dynamics of a 9×9 sheet of compartments under different connectivity schemes. We begin with nearest neighbour coupling, which is a simplistic approximation to the predominance of connectivity between proximal tissue in, for example human cortex.

**Fig. 6:**
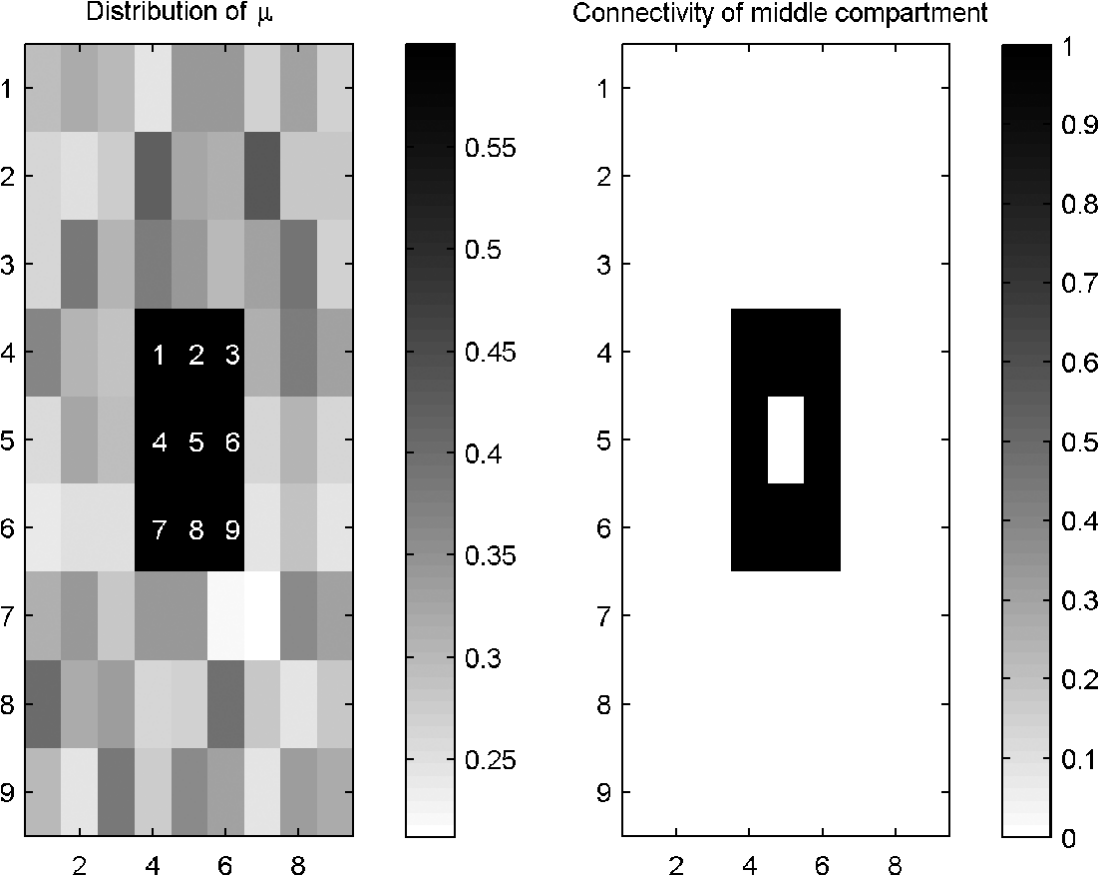
*Schematic of a system with nearest neighbour coupling.* Layout of the 9×9 system of coupled compartments. *Left*: colour coded image of the distribution of *μ*. *The central 9 square*s have μ=0.6, whereas the rest of the system has *μ* drawn from a normal distribution with mean 0.3 and variance 0.05. *Numbers* indicate compartment labels for reference. *Right*: demonstration of connectivity for the central compartment. *Black* indicates the presence of connections

The model can be used to investigate the interplay between network connectivity and the susceptibility of nodes to abnormal states (which is referred to as “epileptogenicity”) [8]. Specifically, higher values of the parameter *μ* can be thought of as being more epileptogenic, since in a single compartment this would render a node closer to the higher amplitude rhythm. As a demonstration of this line of enquiry, a square region of compartments with μ=0.6 is placed at the centre of a nearest neighbour coupled system. This central square is surrounded by “normal” compartments, which are further from the bifurcation and have *μ* drawn from a normal distribution with mean 0.3 and variance 0.05. Figure 6 shows the distribution of *μ* in the system and a visualisation of the connectivity matrix, *A*. 

**Fig. 7:**
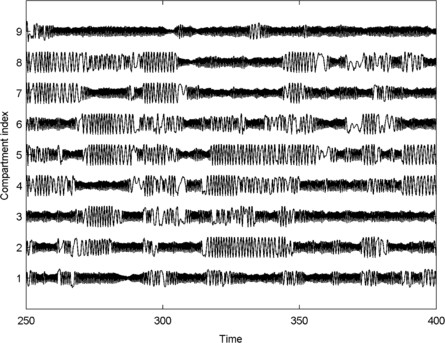
*Dynamics of a system with nearest neighbour coupling.* Time series of the dynamics of the 9 central compartments of the system described in Fig. 6. Compartment numbering is given in Fig. 6. β=2.75

As in the smaller system, *A* was scaled by *β* in order to explore changes in connectivity strength. We found that *β* can be tuned such that compartments of the system undergo intermittent deviations into the high amplitude oscillatory state. An example of the dynamics of the central compartments is given in Fig. 7. It can be seen that different combinations of these compartments undergo periods of high amplitude oscillations. Compartments 1 and 9, at the periphery of the central square are less easily perturbed into the high amplitude state than the other compartments. In particular, compartment 9 is coupled to two compartments with very low values of *μ* (white squares in Fig. 6) and deviates less often into high amplitude rhythms. Thus, it is demonstrated in this system that both the epileptogenicity of nodes and their connected networks is important for state transitions. 

**Fig. 8:**
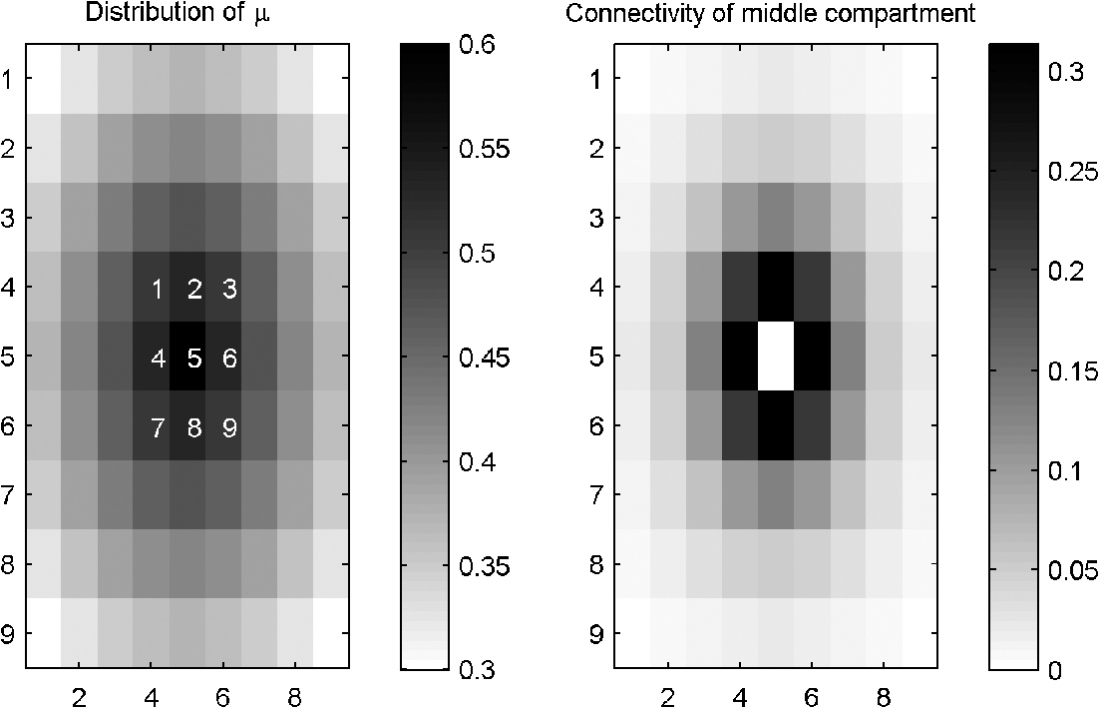
*Schematic of a system with distance dependent coupling.* Layout of the 9×9 system of coupled compartments with distance dependent coupling and exponential decay in *μ*. *Left*: colour coded image of the distribution of *μ*. *The central compartment* has μ=0.6, and the value of *μ* decays exponentially to 0.3 at the periphery of the system. *Numbers* indicate compartment labels for reference. *Right*: colour coded connectivity values for the central compartment

In addition to nearest neighbour coupling, a system with distance dependent connectivity was also explored (see Sect. 2), which more closely approximates short range interactions in the brain. In this system, a graded epileptogenic region was also incorporated by allowing *μ* to decay exponentially from its value of 0.6 in the central compartment to 0.3 at the periphery. The connectivity matrix (*βA*) and the distribution of *μ* for this system can be seen in Fig. 8. 

**Fig. 9:**
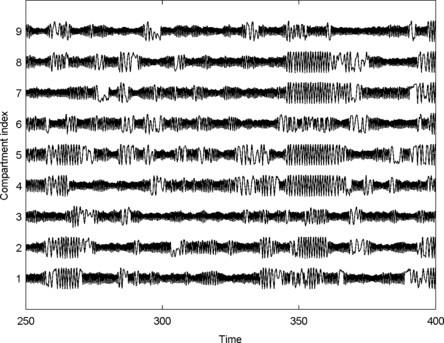
*Dynamics of a system with distance dependent coupling.* Time series of the dynamics of the 9 central compartments of the system described in Fig. 8. Compartment numbering is also provided in Fig. 8. β=5

This system also displayed intermittent high amplitude bursts, as can be seen in Fig. 9. In this case, several bursts were more coherent across the central compartments, even though in this system *μ* varied between compartments with distance from the centre. 

## 4 Discussion

In this paper, we have shown that a network of coupled two-dimensional equations (compartments) can display intermittent behaviour with transitions between low amplitude and high amplitude oscillations. The equations were chosen so that in the absence of coupling the parameters of each compartment were close to a saddle-node bifurcation of periodic orbits creating the large amplitude oscillations, but the different compartments had different parameter values (heterogeneity). This behaviour has also been observed in a more detailed, higher dimensional model of interacting neural masses used as a model of absence epilepsy, so our results suggest that there is an underlying bifurcation mechanism leading to this type of dynamics. This, in turn, shows that the intermittency of the detailed model is robust—it does not depend on the precise terms and parameters of the model. This is important since the model is, by its nature, an approximation. Thus, we have provided strong evidence that these dynamics are due to coupling between heterogeneous systems that posses a region of bistability flanked by saddle-node bifurcations of qualitatively different limit cycles. Furthermore, we confirmed that the bifurcation into the intermittent regime in our reduced model follows the type 1 route [14], which is equivalent to the dynamics of the neural mass model [13]. In addition, we demonstrated the potential of the reduced model to simulate large networks, and hence explore the interplay between network connectivity and spatial distribution of heterogeneities in epileptogenic networks [7,8,16-19]. 

Large scale dynamic models are important for understanding both function and dysfunction in the brain [19-22]. However, as models become larger and more complex, they become difficult to systematically explore, and extraction of underlying principles governing their dynamic repertoire is not trivial. Epilepsy is a pertinent example, where we are challenged to relate multi-scale mechanisms and spatially distributed abnormalities (epileptogenic zones [23] and networks [24]) to the production of pathological phenotypes, which include abnormal electrographic dynamics. Here, we have demonstrated that principles underlying such dynamics can be uncovered by pursuing sequential levels of abstraction. In [7], we formulated a large dimensional neural mass model with the emergent phenomenon of intermittency resembling epileptic electrographic activity. Subsequently in [13], a reduced dimensional neural mass model (i.e. composed of fewer compartments) was shown to preserve this feature and allowed for the categorisation of the route into intermittency at some parameter values as type 1 [14]. In the current study, we have added a further level of abstraction, retaining only certain dynamic features of the original model. 

In terms of brain dynamics, the theoretical prediction of this work is that oscillations in different parts of epileptic brain networks have different degrees of “excitability” due to their proximity to a bifurcation into a different oscillatory dynamic regime. When these regions communicate, e.g. via synaptic connectivity, an intermittent dynamic regime can occur, with spontaneous episodes of qualitatively different dynamics. We have therefore demonstrated a high level theoretical link between brain oscillations [25], differential excitability in brain networks (e.g. [26]) and abnormal rhythm generation, which are crucial concepts for epilepsy. An important next step will be to study models such as the one presented here in order to explore more precisely the relationship between network topology and distributed heterogeneities, which will provide a better understanding of epileptogenic networks and allow to predict the nature of inter-connected normal and abnormal regions of tissue in the epileptic brain [8,16,19]. An advantage of the current framework is that such questions can be explored with improved computational efficiency as compared to neural mass models. 

To confirm the use of our model in this direction, we demonstrated the preservation of intermittency in systems incorporating spatially structured connectivity. Interestingly, in these systems, we did not immediately uncover a dynamic regime in which the majority of nodes displayed concomitant and coordinated switching into abnormal dynamics, as would be expected for absence epilepsy [1]. Our experience simulating spatially structured neural mass models, together with considerations of the anatomy of the brain, suggests that such a regime could require the addition of long range connections. The exploration of this hypothesis will further uncover the link between the topology of large scale brain networks and the propagation of epileptiform activity and will be an interesting avenue for future study. 

An alternative dynamic regime proposed to underly switching between seizure and non-seizure states in absence seizures is noise-driven bistability [4,27-30]. Similarly to our current work, this dynamic regime has been investigated both in mechanistic neural mass models [4] and in reduced forms preserving bistability between a stable steady state and a stable limit cycle [29,30]. In the current study, we employ similar equations, though the resulting bifurcation structure is different as we focus upon transitions between qualitatively different oscillations (following [7]), rather than between a fixed point and a limit cycle. Furthermore, [30] and [29] explored the effect of qualitative changes in network structure on the resulting dynamics. In addition to this work in bistable systems, future such explorations in our low dimensional framework for intermittency can provide insight into the role of network connectivity on seizure occurrence. In addition, recent work has explored the possibility that critical transitions are responsible for seizure termination in focal onset seizures with secondary generalisation. [11] showed that certain features of these transitions, such as slowing and flickering [31] can be seen in recordings from patients with these seizure types, and slowing of absence seizure rhythms toward seizure termination does occur. In our intermittent system, the dynamics of seizure termination are governed by the nature of the vector field where the system leaves the previously stable limit cycle. Whether such hallmarks can be seen in intermittent models is yet to be determined. Although in the current study we focused on robustness of the mechanisms of intermittent transitions, in future studies, it will be interesting to explore other details of the waveforms of seizure EEG. 

A similar model approximation approach was taken recently by [32], who sought to uncover the dynamic principles underlying multi-stability and scale invariant fluctuations in the human alpha rhythm. Similarly to the current study, their starting point was a biophysically inspired neural mass model which offered a novel explanation for the observed data [33]. Studying a normal form for the dynamics of interest, the requirements were shown to be a sub-critical Hopf bifurcation and the incorporation of multiplicative noise. Other abstract modelling studies have demonstrated minimal neural mass models underlying the generation of different epileptic electroencephalographic waveforms [34], as well as the complex role of networks underlying focal and generalised epileptic seizures [17]. The approach of modelling at several levels of abstraction will be a key strategy in advancing our understanding of the brain. 

In summary, we have uncovered a dynamic mechanism responsible for spontaneous state transitions in a neural mass model of epilepsy. Future explorations of this model and further developments and applications of the general methods employed herein will help to advance our understanding of large scale brain dynamics in health and disease. Since our model is framework independent, it might also be used to explore rhythm generation in other systems, for example oscillating biochemical networks [35]. 

## Competing Interests

The authors declare that they have no competing interests.

## Authors’ Contributions

MG and PG designed the study, performed analysis and wrote the paper. All authors read and approved the final manuscript.
